# Supplementary material for: Bioactive Compounds and Pharmacological Properties of the Polypore Fomes fomentarius, a Medicinal Wild Mushroom Collected from Morocco
Source: Int J Mol Sci. 2025 Sep 21;26(18):9215. doi: 10.3390/ijms26189215 (PMC12470752; doi:10.3390/ijms26189215)
Supplement: Supplementary file 1 [file ijms-26-09215-s001.zip › ijms-3848017-supplementary.pdf]

International Journal of *Molecular Sciences*

*Supplementary Materials*

# Bioactive Compounds and Pharmacological Properties of the Polypore *Fomes fomentarius*, a Medicinal Wild Mushroom Collected from Morocco

**El Hadi Erbiai [1,2,3,*] Safae Maouni [2,4], Luís Pinto da Silva [3], Rabah Saidi [2], Zouhaire Lamrani [2], Joaquim C. G. Esteves da Silva [3], Abdelfettah Maouni [2], Eugénia Pinto [5,6,*]**

[1] Centre for the Research and Technology of Agroenvironmental and Biological Sciences, CITAB, Inov4Agro, Universidadede Trás-os-Montes e Alto Douro, UTAD, Quinta de Prados, 5000-801 Vila Real, Portugal
[2] Biology, Environment, and Sustainable Development Laboratory, École Normale Supérieure de Tétouan (ENS), Abdelmalek Essaadi University, Tetouan 93000, Morocco; maouni.safae88@gmail.com (S.M.); r.saidi@uae.ac.ma (R.S.);
zh.amrani@yahoo.fr (Z.L.); amaouni@uae.ac.ma (A.M.)
[3] Chemistry Research Unit (CIQUP), Institute of Molecular Sciences (IMS), Department of Geosciences, Environment and Spatial Plannings, Faculty of Sciences, University of Porto, Rua do Campo Alegre s/n, 4169-007 Porto, Portugal; luis.silva@fc.up.pt (L.P.d.S.); jcsilva@fc.up.pt (J.C.G.E.d.S.)
[4] Department of Dermatology, Mohamed 5 University, B.P. 6527, Rabat, Morocco
[5] Laboratory of Microbiology, Biological Sciences Department, Faculty of Pharmacy, University of Porto, 4050-313 Porto, Portugal
[6] Interdisciplinary Centre of Marine and Environmental Research (CIIMAR), University of Porto, 4450-208 Matosinhos, Portugal
* Correspondence: eherbiai@utad.pt(E.H.E.); epinto@ff.up.pt (E.P.)

Academic Editor: Stefania Garzoli

Received: 14 August 2025
Revised: 13 September 2025
Accepted: 19 September 2025
Published: date

**Citation:** Erbiai, E.H.; Maouni, S.; Pinto da Silva, L.; Saidi, R.; Lamrani, Z.; Esteves da Silva, J.C.G.; Maouni, A.; Pinto, E. Bioactive Compounds and Pharmacological Properties of the Polypore *Fomes fomentarius*, a Medicinal Wild Mushroom Collected from Morocco. *Int. J. Mol. Sci.* **2025**, *26*, x. https://doi.org/10.3390/xxxxx

**Copyright:** © 2025 by the authors. Submitted for possible open access publication under the terms and conditions of the Creative Commons Attribution (CC BY) license (https://creativecommons.org/licenses/by/4.0/).

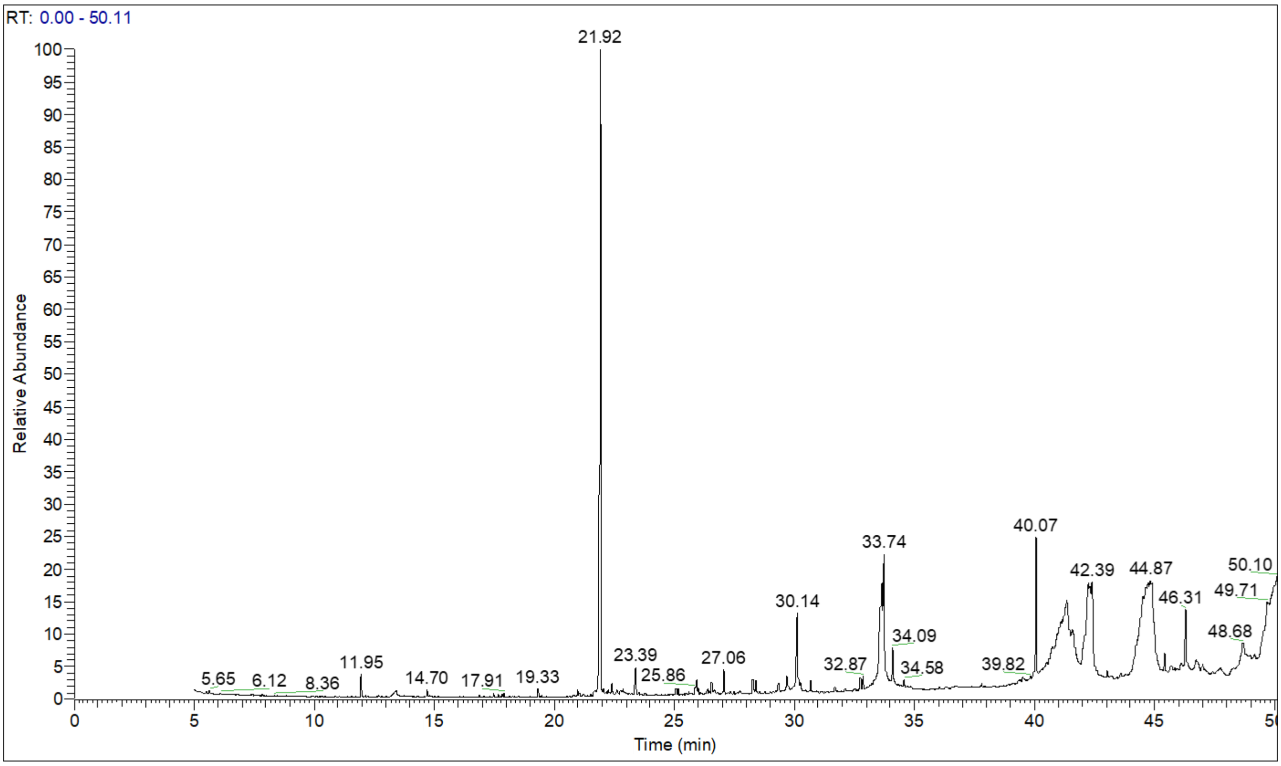


**Figure S1**. GC-MS chromatogram of the biomolecules profile of *F. fomentarius* methanolic extract diluted in chloroform

**Table S1.** Phenols identified by GC-MS in *F. fomentarius* methanolic extract diluted in chloroform.

| Compound names | Chemical formula | Molecular weight | Retention time | Kovats' RI | Area % |
|---|---|---|---|---|---|
| 2,4-Diisopropyl-5-methylphenol | $C_{13}H_{20}O$ | 192.3 | 25.94 | 1463.84 | 0.43 |
| **2,6-Di-tert-butyl-4-(dimethylaminomethyl)phenol** | $C_{17}H_{29}NO$ | **263.4** | **41.34** | **2234.81** | **18.16** |
| **4-(3,5-Di-tert-butyl-4-hydroxyphenyl)butyl acrylate** | $C_{21}H_{32}O_3$ | **332.5** | **44.86** | **2451.25** | **21.71** |

**Table S2.** Alcohols identified by GC-MS in the *F. fomentarius* methanolic extract diluted in chloroform.

| Compound names | Chemical formula | Molecular weight | Retention time | Kovats' RI | Area % |
|---|---|---|---|---|---|
| 2-Butyl-1-octanol | $C_{12}H_{26}O$ | 186.33 | 17.48 | 1102.37 | 0.15 |
| 2-Hexyldodecanol | $C_{18}H_{38}O$ | 270.5 | 22.07 | 1188.70 | 0.15 |
| 2-Hexyldodecyl acetate | $C_{20}H_{40}O_2$ | 312.5 | 22.61 | 1312.05 | 0.22 |
| Hexadecen-1-ol, trans-9- | $C_{16}H_{32}O$ | 240.42 | 27.07 | 1518.09 | 0.33 |
| 1-Heneicosanol | $C_{21}H_{44}O$ | 312.6 | 30.68 | 1694.11 | 0.21 |

**Table S3.** Alkaloids identified by GC-MS in *F. fomentarius* methanolic extract diluted in chloroform.

| Compound names | Chemical formula | Molecular weight | Retention time | Kovats' RI | Area % |
|---|---|---|---|---|---|
| Perivine | $C_{20}H_{22}N_2O_3$ | 338.4 | 46.31 | 2516.72 | 1.68 |
| **5-Benzylquinoline** | $C_{16}H_{13}N$ | **219.28** | **50.1** | **2640** | **9.87** |

**Table S4.** Fatty acids identified by GC-MS in *F. fomentarius* methanolic extract diluted in chloroform.

| Compound names | Chemical formula | Molecular weight | Retention time | Kovats' RI | Area % |
|---|---|---|---|---|---|
| Lauric Acid | $C_{12}H_{24}O_2$ | 200.32 | 22.88 | 1324.10 | 0.22 |
| Myristic acid | $C_{14}H_{28}O_2$ | 228.37 | 26.56 | 1492.95 | 0.19 |
| Pentadecanoic acid | $C_{15}H_{30}O2$ | 242.4 | 28.27 | 1578.39 | 0.23 |
| *i*-Propyl 15-methylhexadecanoate | $C_{20}H_{40}O_2$ | 312.5 | 29.35 | 1630.80 | 0.21 |
| Palmitoleic acid | $C_{16}H_{30}O_2$ | 254.41 | 29.7 | 1647.39 | 0.67 |
| **Palmitic Acid** | $\mathbf{C_{16}H_{32}O_2}$ | **256.42** | **30.13** | **1667.77** | **3.01** |
| *cis*-10-Heptadecenoic acid | $C_{17}H_{32}O_2$ | 268.4 | 31.69 | 1739.81 | 0.09 |
| Linoleic acid methyl ester | $C_{19}H_{34}O_2$ | 294.5 | 32.75 | 1787.78 | 0.2 |
| *trans*-13-Octadecenoic acid, methyl ester | $C_{19}H_{36}O_2$ | 296.48 | 32.87 | 1793.21 | 0.21 |
| **Linoelaidic acid** | $\mathbf{C_{18}H_{32}O_2}$ | **280.4** | **33.64** | **1830.09** | **5.28** |
| ***cis*-Vaccenic acid** | $\mathbf{C_{18}H_{34}O_2}$ | **282.5** | **33.74** | **1834.95** | **4.21** |
| Stearic acid | $C_{18}H_{36}O_2$ | 284.5 | 34.09 | 1851.94 | 1.28 |

**Table S5.** Alkanes identified by GC-MS in the *F. fomentarius* methanolic extract diluted in chloroform.

| Compound names | Chemical formula | Molecular weight | Retention time | Kovats' RI | Area % |
|---|---|---|---|---|---|
| Hexachloroethane | $C_2Cl_6$ | 236.7 | 11.95 | 908.11 | 0.39 |
| 5,8-Diethyldodecane | $C_{16}H_{34}$ | 226.44 | 21.7 | 1273.22 | 0.18 |
| 1,3,5-Trimethyl-2-octadecylcyclohexane | $C_{27}H_{54}$ | 378.7 | 22.26 | 1296.65 | 0.12 |
| 5-Methyltetradecane | $C_{15}H_{32}$ | 212.41 | 22.4 | 1302.67 | 0.24 |
| Pentadecane | $C_{15}H_{32}$ | 212.4 | 22.77 | 1319.19 | 0.18 |
| Cyclohexadecane | $C_{16}H_{32}$ | 224.42 | 23.39 | 1346.87 | 0.34 |
| Heneicosane | $C_{21}H_{44}$ | 296.6 | 34.33 | 1863.59 | 0.21 |

**Table S6.** Other groups of biomolecules identified by GC-MS in *F. fomentarius* methanolic extract diluted in chloroform.

| Compound names | Chemical formula | Molecular weight | Retention time | Kovats' RI | Area % | Groups |
|---|---|---|---|---|---|---|
| 2-Methyl-4-oxopentanoic acid | $C_6H_{10}O_3$ | 130.139 | 13.42 | 955.84 | 0.5 | Organic acid |
| Naphthalene | $C_{10}H_8$ | 128.169 | 14.7 | 997.40 | 0.13 | Aromatic hydrocarbon |
| 1-Tetradecene | $C_{14}H_{28}$ | 196.37 | 19.33 | 1175.49 | 0.13 | Alkene |
| 2,6-Diisopropylnaphthalene | $C_{16}H_{20}$ | 212.33 | 25.08 | 1423.47 | 0.07 | Aromatic hydrocarbon |
| Phthalic acid, hex-3-yl isobutyl ester | $C_{18}H_{26}O_4$ | 306.4 | 28.41 | 1585.42 | 0.18 | Ester |
| **2-(2,4-Di-tert-pentylphenoxy)butanoic acid** | $\mathbf{C_{20}H_{32}O_3}$ | **320.5** | **41.58** | **2250** | **2.59** | **Organic acid** |
| **Diisooctyl phthalate** | $\mathbf{C_{24}H_{38}O_4}$ | **390.6** | **40.07** | **2156.62** | **2.37** | **Ester** |
| **Betulin** | $\mathbf{C_{30}H_{50}O_2}$ | **442.7** | **42.4** | **2302.04** | **10** | **Triterpenoid** |
| Dehydroergosterol | $C_{28}H_{42}O$ | 394.6 | 45.43 | 2479.89 | 0.31 | Steroid |

**Table S7.** Sugar compositions identified by GC-MS analysis in derivatized *F. fomentarius* methanolic extract.

| | Compound names | Chemical formula | Molecular weight | Kovats' RI* | Area % |
|---|---|---|---|---|---|
| Monosaccharides | 2-Deoxy-erythro-pentonic acid | $C_5H_{10}O_5$ | 150.13 | 1667.64 | 0.1 |
| | $\alpha$-D-lyxopyranose | $C_5H_{10}O_5$ | 150.13 | 1620.09 | 0.4 |
| | $\beta$-D-allopyranose | $C_6H_{12}O_6$ | 180.16 | 1829.29 | 2.42 |
| | $\beta$-D-mannopyranose | $C_6H_{12}O_6$ | 180.16 | 1934.89 | 0.97 |
| | $\beta$-D-ribopyranose | $C_5H_{10}O_5$ | 150.13 | 1629.41 | 0.71 |
| | $\beta$-D-talopyranose | $C_6H_{12}O_6$ | 180.16 | 1930.72 | 0.86 |
| | $\beta$-D-xylopyranose | $C_5H_{10}O_5$ | 150.13 | 1671.07 | 0.1 |
| | $\beta$-L-fucopyranose | $C_6H_{12}O_5$ | 164.16 | 1690.19 | 0.22 |
| | D-galactopyranose | $C_6H_{12}O_6$ | 180.16 | 1882.82 | 0.59 |
| | D-gluconic acid | $C_6H_{12}O_7$ | 196.16 | 2037.11 | 1.46 |
| | **D-glucopyranose** | **$C_6H_{12}O_6$** | **180.16** | **1918.75** | **14.64** |
| | **D-mannitol** | **$C_6H_{14}O_6$** | **182.17** | **1959.89** | **9.75** |
| | D-pinitol | $C_7H_{14}O_6$ | 194.18 | 1858.58 | 0.78 |
| | Erythritol | $C_4H_{10}O_4$ | 122.12 | 1514.92 | 0.54 |
| | Glyceric acid | $C_3H_6O_4$ | 106.08 | 1332.79 | 0.08 |
| | Glycerol | $C_3H_8O_3$ | 92.09 | 1275 | 2.55 |
| | *Myo*-inositol | $C_6H_{12}O_6$ | 180.16 | 2120.68 | 2.76 |
| | N-acetylglucosamine | $C_8H_{15}NO_6$ | 221.20 | 2130.45 | 0.4 |
| | **Xylitol** | **$C_5H_{12}O_5$** | **152.15** | **1738.8** | **11.62** |
| Disaccharides | **3-$\alpha$-mannobiose** | **$C_{12}H_{22}O_{11}$** | **342.3** | **2735.22** | **2.46** |
| | **D-trehalose** | **$C_{12}H_{22}O_{11}$** | **342.3** | **2802.95** | **8.07** |
| | **Maltitol** | **$C_{12}H_{22}O_{11}$** | **342.3** | **2974.27** | **2.09** |
| | Maltose | $C_{12}H_{22}O_{11}$ | 342.3 | 2702.84 | 0.79 |
| | Melibiose | $C_{12}H_{22}O_{11}$ | 342.3 | 2956.84 | 0.27 |

*Kovats' RI: Kovats retention index of compound's derivative form.

**Table S8.** Fatty acids identified by GC-MS analysis in derivatized *F. fomentarius* methanolic extract.

| Compound names | Chemical formula | Molecular weight | Kovats' RI | Area % |
|---|---|---|---|---|
| **4-Oxohexanoic acid** | **$C_6H_{10}O_3$** | **130.139** | **1159.03** | **2.64** |
| 10,12-Docosadiynedioic acid | $C_{22}H_{34}O_4$ | 362.5 | 2420.88 | 0.22 |
| 5,8,11,14-Eicosatetraynoic acid | $C_{20}H_{24}O_2$ | 296.4 | 3246.22 | 0.2 |
| Azelaic acid | $C_9H_{16}O_4$ | 188.22 | 1802.02 | 0.33 |
| **Elaidic acid** | **$C_{18}H_{34}O_2$** | **282.5** | **2224.26** | **3.35** |
| Heptanoic acid | $C_7H_{14}O_2$ | 130.18 | 2638.6 | 0.74 |
| **Linoleic acid** | **$C_{18}H_{32}O_2$** | **280.4** | **2219.52** | **3.81** |
| Oleic acid | $C_{18}H_{34}O_2$ | 282.5 | 2228.4 | 0.38 |
| **Palmitic acid** | **$C_{16}H_{32}O_2$** | **256.42** | **2048.96** | **1.98** |
| Palmitoleic acid | $C_{16}H_{30}O_2$ | 254.41 | 2032.98 | 0.36 |
| Stearic acid | $C_{18}H_{36}O_2$ | 284.5 | 2244.37 | 0.71 |

**Table S9.** Organic acids identified by GC-MS analysis in derivatized *F. fomentarius* methanolic extract.

| Compound names | Chemical formula | Molecular weight | Kovats' RI | Area % |
|---|---|---|---|---|
| Acetylpyruvic acid | $C_5H_6O_4$ | 130.1 | 1297.72 | 0.72 |
| Citric acid | $C_6H_8O_7$ | 192.12 | 1837.37 | 0.85 |
| Fumaric acid | $C_4H_4O_4$ | 116.07 | 1345.16 | 0.15 |
| Lactic acid | $C_3H_6O_3$ | 90.08 | 1066.45 | 0.67 |
| **Malic acid** | **$C_4H_6O_5$** | **134.09** | **1493.87** | **1.87** |
| Phthalic acid | $C_8H_6O_4$ | 166.13 | 2567.1 | 0.88 |
| Succinic acid | $C_4H_6O_4$ | 118.09 | 1313.97 | 0.22 |

**Table S10.** Steroids identified by GC-MS analysis in derivatized *F. fomentarius* methanolic extract.

| Compound names | Chemical formula | Molecular weight | Kovats' RI | Area % |
|---|---|---|---|---|
| **Ergosta-7,22-dien-3β-ol** | **$C_{28}H_{46}O$** | **398.7** | **3285.81** | **1.43** |
| **Ergosterol** | **$C_{28}H_{44}O$** | **396.6** | **3267.96** | **2.27** |
| Glycocholic acid | $C_{26}H_{43}NO_6$ | 465.6 | 3042.85 | 0.06 |

**Table S11.** Alcohols identified by GC-MS analysis in derivatized *F. fomentarius* methanolic extract.

| Compound names | Chemical formula | Molecular weight | Kovats' RI | Area % |
|---|---|---|---|---|
| 1,2-Octadecanediol | $C_{18}H_{38}O_2$ | 286.5 | 2399.393 | 0.34 |
| 2,3-Butanediol | $C_4H_{10}O_2$ | 90.12 | 1040.37 | 0.26 |
| *β*-Eudesmol | $C_{15}H_{26}O$ | 222.37 | 2211.83 | 0.1 |
| Diethylene glycol | $C_4H_{10}O_3$ | 106.12 | 1244.318 | 0.73 |
| **Propylene glycol** | **$C_3H_8O_2$** | **76.09** | **1009.31** | **1.84** |

**Table S12.** Amino acids identified by GC-MS analysis in derivatized *F. fomentarius* methanolic extract.

| Compound names | Chemical formula | Molecular weight | Kovats' RI | Area % |
|---|---|---|---|---|
| Alanine | $C_3H_7NO_2$ | 89.09 | 1107.83 | 0.2 |
| **$\gamma$-Aminobutyric acid** | **$C_4H_9NO_2$** | **103.12** | **1538.8** | **0.39** |
| Glycine | $C_2H_5NO_2$ | 75.07 | 1055.9 | 0.1 |
| Proline | $C_5H_9NO_2$ | 115.13 | 1306.99 | 0.13 |
| Threonine | $C_4H_9NO_3$ | 119.12 | 1391.94 | 0.07 |
| Valine | $C_5H_{11}NO_2$ | 117.15 | 1220.45 | 0.19 |

**Table S13.** Other biomolecule groups identified by GC-MS analysis in derivatized *F. fomentarius* methanolic extract.

| Compound names | Chemical formula | Molecular weight | Kovats' RI | Area % | Groups |
|---|---|---|---|---|---|
| 3,4,3',4'-Tetrahydrospirilloxanthin | $C_{42}H_{64}O_2$ | 601 | 3207.32 | 0.83 | Carotenoids |
| 5-Methyluridine | $C_{10}H_{14}N_2O_6$ | 258.23 | 2436.7 | 0.15 | Nucleosides |
| **Adenosine** | $\mathbf{C_{10}H_{13}N_5O_4}$ | **267.24** | **2669.62** | **1.36** | **Nucleosides** |
| Uridine | $C_9H_{12}N_2O_6$ | 244.2 | 2487.34 | 0.26 | Nucleosides |
| **1-Octadecene** | $\mathbf{C_{18}H_{36}}$ | **252.5** | **1796.01** | **1.24** | **Alkenes** |
| Gentisic acid | $C_7H_6O_4$ | 154.12 | 1790.04 | 0.14 | Phenol |
| **Phosphoric acid** | $\mathbf{H_3O_4P}$ | **97.99** | **1280.68** | **1.57** | **Organosilicon compound** |
| Dodecamethylpentasiloxane | $C_{12}H_{36}O_4Si_5$ | 384.84 | 1172.89 | 0.19 | Organosilicon compound |
| Octamethyltrisiloxane | $C_8H_{24}O_2Si_3$ | 236.53 | 900.63 | 0.59 | Organosilicon compound |
| Glycerophosphoric acid | $C_3H_9O_6P$ | 172.07 | 1779.6 | 0.21 | Glycerophosphates |
| Glyceryl-glycoside | $C_9H_{18}O_8$ | 254.23 | 2254.54 | 0.16 | Glyceroglycolipids |
| 2-Monoolein | $C_{21}H_{40}O_4$ | 356.5 | 2980.91 | 0.13 | Glycerolipids |
| Acetone | $C_3H_6O$ | 58.08 | 958.22 | 0.25 | Oxygenated hydrocarbon |
| Ethanolamine | $C_2H_7NO$ | 61.08 | 1325.26 | 0.09 | Amine |

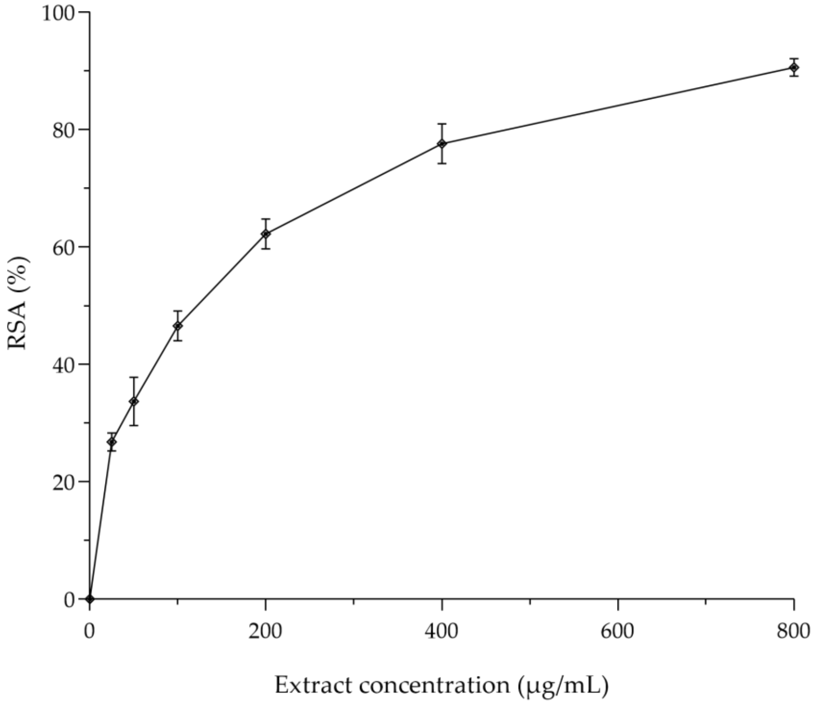


**Figure S2.** Percentage of DPPH radical-scavenging activity (RSA) of *F. fomentarius* methanolic extract. Values are mean ± standard deviation ($n = 3$)

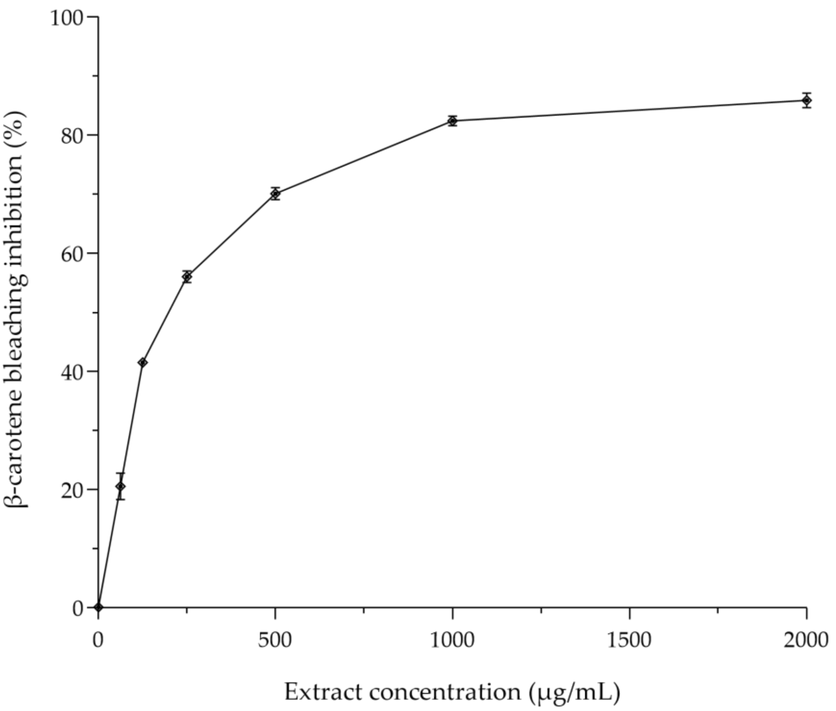


**Figure S3.** Percentage of the β-carotene bleaching inhibition of *F. fomentarius* methanolic extract. Values are mean ± standard deviation ($n = 3$)

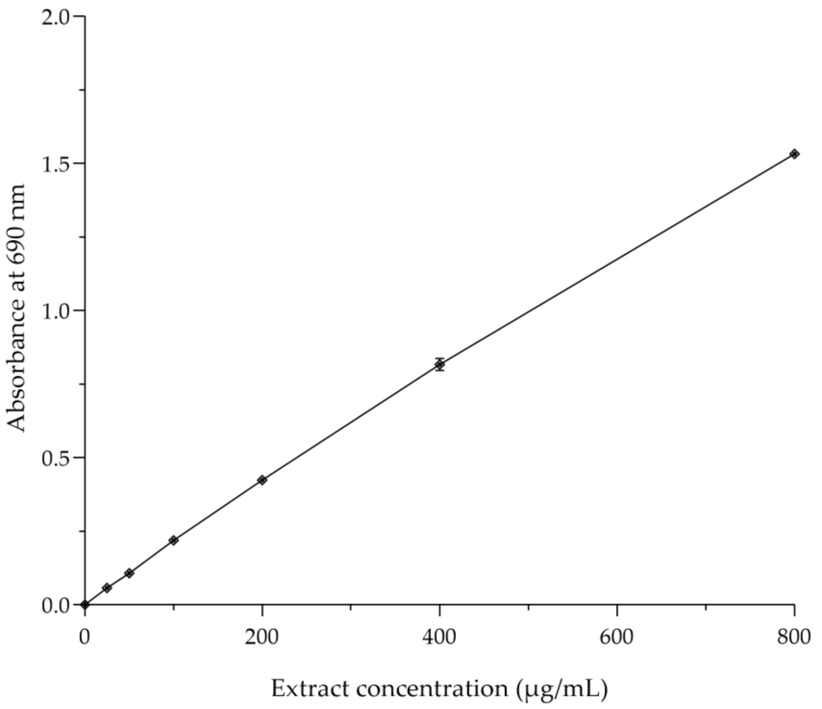


**Figure S4.** Ferric ion-reducing power of *F. fomentarius* methanolic extract. Values are mean ± standard deviation ($n = 3$)
